# Increased Glutathione Synthesis Following Nrf2 Activation by Vanadyl Sulfate in Human Chang Liver Cells

**DOI:** 10.3390/ijms12128878

**Published:** 2011-12-05

**Authors:** Areum Daseul Kim, Rui Zhang, Kyoung Ah Kang, Ho Jin You, Jin Won Hyun

**Affiliations:** 1 School of Medicine, Jeju National University, Jeju 690-756, Korea; E-Mails: candy4860@hanmail.net (A.D.K.); zhangrui26@hotmail.com (R.Z.); 2 Division of Radiation Cancer Research, Korea Institute of Radiological and Medical Sciences, Seoul 139-706, Korea; E-Mail: legna48@hanmail.net; 3 Department of Biomaterials, DNA Repair Center, Chosun University, Gwangju 501-759, Korea; E-Mail: hjyou@chosun.ac.kr

**Keywords:** Jeju ground water, vanadyl sulfate, glutamate cysteine ligase, human Chang liver cells, erythroid transcription factor NF-E2

## Abstract

Jeju ground water, containing vanadium compounds, was shown to increase glutathione (GSH) levels as determined by a colorimetric assay and confocal microscopy. To investigate whether the effects of Jeju ground water on GSH were specifically mediated by vanadium compounds, human Chang liver cells were incubated for 10 passages in media containing deionized distilled water (DDW), Jeju ground water (S1 and S3), and vanadyl sulfate (VOSO_4_). Vanadyl sulfate scavenged superoxide anion, hydroxyl radical and intracellular reactive oxygen species. Vanadyl sulfate effectively increased cellular GSH level and up-regulated mRNA and protein expression of a catalytic subunit of glutamate cysteine ligase (GCLC), which is involved in GSH synthesis. The induction of GCLC expression by vanadyl sulfate was found to be mediated by transcription factor erythroid transcription factor NF-E2 (Nrf2), which critically regulates GCLC by binding to the antioxidant response elements (AREs). Vanadyl sulfate treatment increased the nuclear translocation of Nrf2 and the accumulation of phosphorylated Nrf2. Extracellular regulated kinase (ERK) contributed to ARE-driven GCLC expression via Nrf2 activation. Vanadyl sulfate induced the expression of the active phospho form of ERK. Taken together, these results suggest that the increase in GSH level by Jeju ground water is, at least in part, due to the effects of vanadyl sulfate via the Nrf2-mediated induction of GCLC.

## 1. Introduction

Reduced glutathione (GSH) is an abundant intracellular thiol peptide found in aerobic cells that is involved in cellular detoxification, antioxidant defenses, the maintenance of thiol status, and the modulation of cell proliferation [1]. Intracellular GSH concentrations are influenced by multiple factors and reflect a balance between rates of consumption/efflux and *de novo* synthesis. The latter proceeds by two ATP-requiring enzymatic steps: the formation of γ-glutamylcysteine from glutamate and cysteine, and then GSH from γ-glutamylcysteine and glycine. The first step of GSH biosynthesis is rate-limiting and catalyzed by glutamate cysteine ligase (GCL), a heterodimer composed of a catalytic subunit (GCLC, 73 kDa) and a modulatory subunit (GCLM, 29 kDa) [2]. The second step of GSH synthesis is catalyzed by glutathione synthetase, a homodimer of 52 kDa. Since GSH is crucial to cellular antioxidant defense against oxidant injury, the induction of these regulatory enzymes is a key step in the defense mechanism. GCLC is up-regulated through antioxidant-response elements (AREs), which are also known as electrophile-responsive elements [3,4]. The consensus ARE core sequence shows remarkable similarity to the binding sequence of the erythroid transcription factor NF-E2 (Nrf2). Accumulated evidence indicates the involvement of Nrf2 in the regulation of GCLC gene expression [4,5].

Recently, we reported that Jeju ground water possesses *in vitro* and *in vivo* antioxidant effects [6–8] as well as immune-stimulating properties, as determined in the peripheral immunocytes of γ-irradiated mice [9]. Jeju ground water contains trace amounts of vanadium compounds, which have been shown to exert antioxidant effects [10–12].

In the present study, to investigate whether the effects of Jeju ground water on GSH are specifically mediated by vanadium compounds, GSH synthesis and related mechanisms were studied in human Chang liver cells exposed to vanadyl sulfate (VOSO_4_).

## 2. Results and Discussion

### 2.1. GSH Amount Is Enhanced by Jeju Ground Water Containing Vanadium Components

**Figure 1:**
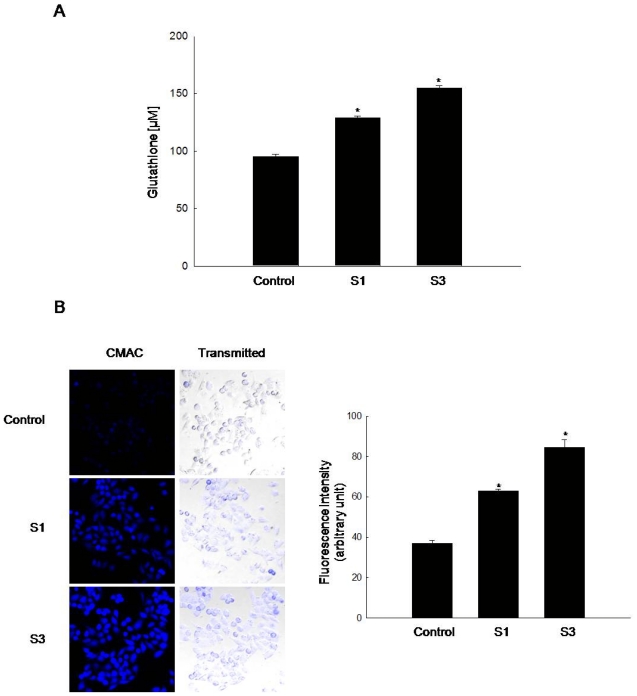
Jeju ground water increases cellular glutathione (GSH) level. Cells were incubated for 10 passages in media containing deionized distilled water (DDW) (control) and Jeju ground water (S1 and S3). Cellular GSH level was detected using (**A**) a colorimetric assay kit and (**B**) confocal microscopy after tert-butoxycarbonyl-Leu-Met-7-amino-4-chloromethylcoumarine (CMAC) staining and quantified. * Significantly different from the control group (*P* < 0.05).

GSH is a cellular sulfhydryl-containing molecule responsible for maintaining cellular oxidation-reduction homeostasis. Alterations in GSH level can be monitored and serve as an indicator of oxidative stress [13]. GSH level was measured in human Chang liver cells cultured with Jeju ground water preparations S1 and S3, containing vanadium compounds at concentrations of 8.0 ± 0.9 and 26.0 ± 2.0 μg/L, respectively. After 10 passages, S1- and S3-cultured cells showed statistically significant increases in cellular GSH level of 129 and 155 μM, respectively, compared to 95 μM in DDW-cultured cells (control), as determined in the colorimetric assay (Figure 1A). Confocal microscopy showed an increase in blue fluorescence intensity of cellular GSH in S1- and S3-treated cells compared to DDW-treated cells (control) (Figure 1B). S3-treated cells showed an increase in GSH levels compared to S1-treated cells (Figure 1A) and the fluorescence intensity of cellular GSH was also enhanced in S3-treated cells compared to S1-treated cells (Figure 1B). Thus S3 ground water showed more effective in induction of GSH levels compared to S1 ground water.

### 2.2. Radical Scavenging Activity of Vanadyl Sulfate

**Figure 2:**
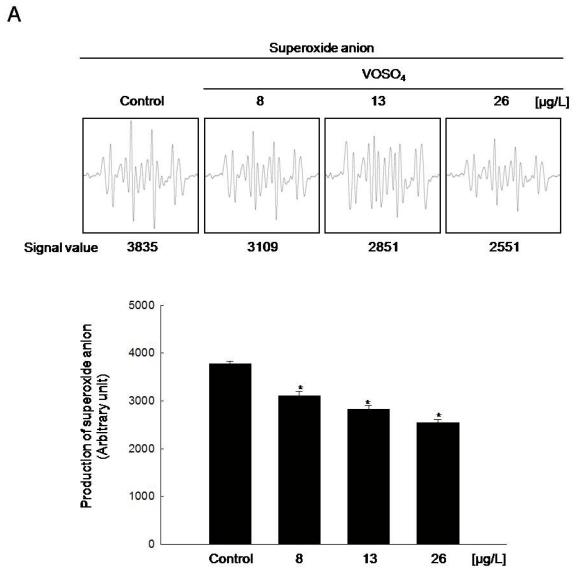

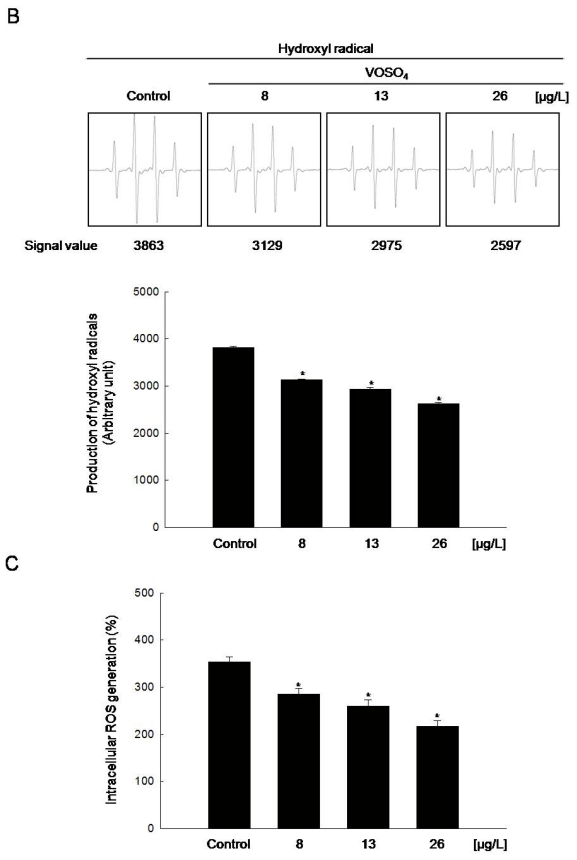

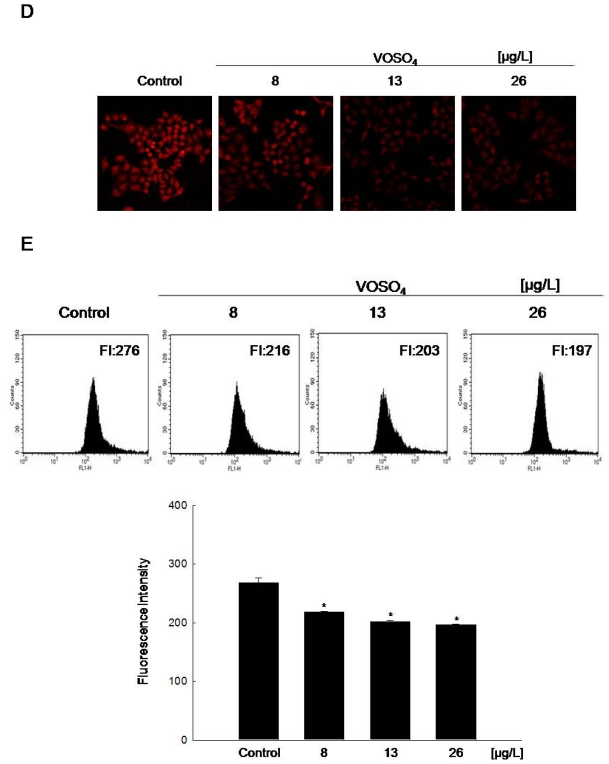
Vanadyl sulfate scavenges ROS. (**A**) Superoxide anion generated by xanthine and xanthine oxidase was reacted with 5,5-dimethyl-1-pyrroline-*N*-oxide (DMPO), and the resultant DMPO/·OOH adducts were detected by electron spin resonance (ESR) spectrometry; (**B**) Hydroxyl radical generated by the Fenton reaction (H_2_O_2_ + FeSO_4_) was reacted with DMPO and the resultant DMPO/·OH adducts were detected by ESR spectrometry. Cells were incubated for 10 passages in media containing DDW (control) and VOSO_4_; Intracellular ROS were detected using (**C**) fluorescence spectrophotometry; (**D**) confocal microscopy, and (**E**) flow cytometry after 2′,7′-dichlorodihydrofluorescein diacetate (DCF-DA) staining. * Significantly different from the control group (*P* < 0.05).

In a recent report, we described the ROS scavenging effects of S1 and S3 [6]. To investigate whether these effects are due to the presence of vanadium compounds, the ability of vanadyl sulfate to scavenge superoxide anion and hydroxyl radical was measured by electron spin resonance (ESR) spectrometry. An average increase in the superoxide anion signal to a value of 3781 in the xanthine/xanthine oxidase system was determined in cells exposed to DDW (control) whereas vanadyl sulfate treatment decreased the superoxide anion signal to 3102, 2826, and 2550 at VOSO_4_ concentrations of 8, 13, and 26 μg/L, respectively (Figure 2A). Consistent with its superoxide anion-scavenging activity, vanadyl sulfate treatment also reduced hydroxyl radical generation by the Fenton reaction (H_2_O_2_ + FeSO_4_) to average values of 3129, 2932, and 2631 at VOSO_4_ concentrations of 8, 13, and 26 μg/L, respectively, compared to 3814 in DDW treatment (control) (Figure 2B). These results suggested that vanadyl sulfate is a free-radical scavenger. The intracellular ROS scavenging ability of vanadyl sulfate in human Chang liver cells was also measured. In H_2_O_2_-treated cells with DDW, intracellular ROS generation increased by 354% whereas vanadyl sulfate treatment reduced intracellular ROS by 286%, 260%, and 217% at VOSO_4_ concentrations of 8, 13, and 26 μg/L, respectively (Figure 2C). Confocal microscopy analysis likewise revealed that vanadyl sulfate treatment reduced the red fluorescence intensity of H_2_O_2_-induced ROS compared to DDW treatment (control), as shown in Figure 2D. In addition, ROS level detected by flow cytometry yielded average fluorescence intensity value of 218, 201, and 197 in H_2_O_2_-treated cells with 8, 13, and 26 μg/L of VOSO_4_, respectively, compared to a value of 268 in H_2_O_2_-treated cells with DDW (Figure 2E). Thus, VOSO_4_ at 8, 13, and 26 μg/L was sufficient to effectively inhibit intracellular ROS. This intracellular ROS-scavenging activity of vanadyl sulfate is consistent with the ESR data. We also determined the effect of VOSO_4_ at 52 μg/L on free radicals scavenging activity. VOSO_4_ at 52 μg/L diminished the scavenging effect of ROS (data not shown). Taken together, these results suggest that the antioxidant effects of vanadyl sulfate are mediated by ROS scavenging.

### 2.3. Induction of GSH Synthesis by Vanadyl Sulfate

**Figure 3:**
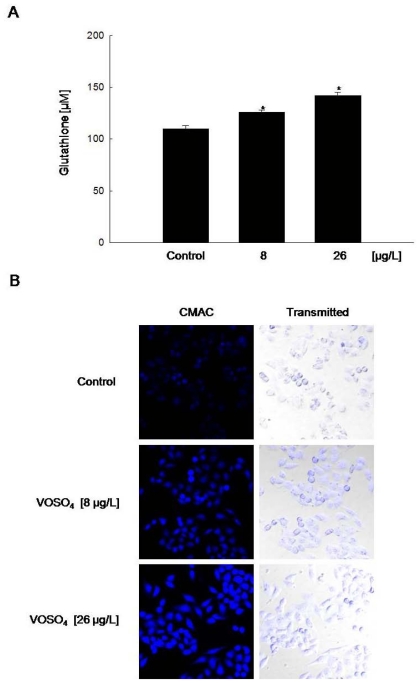

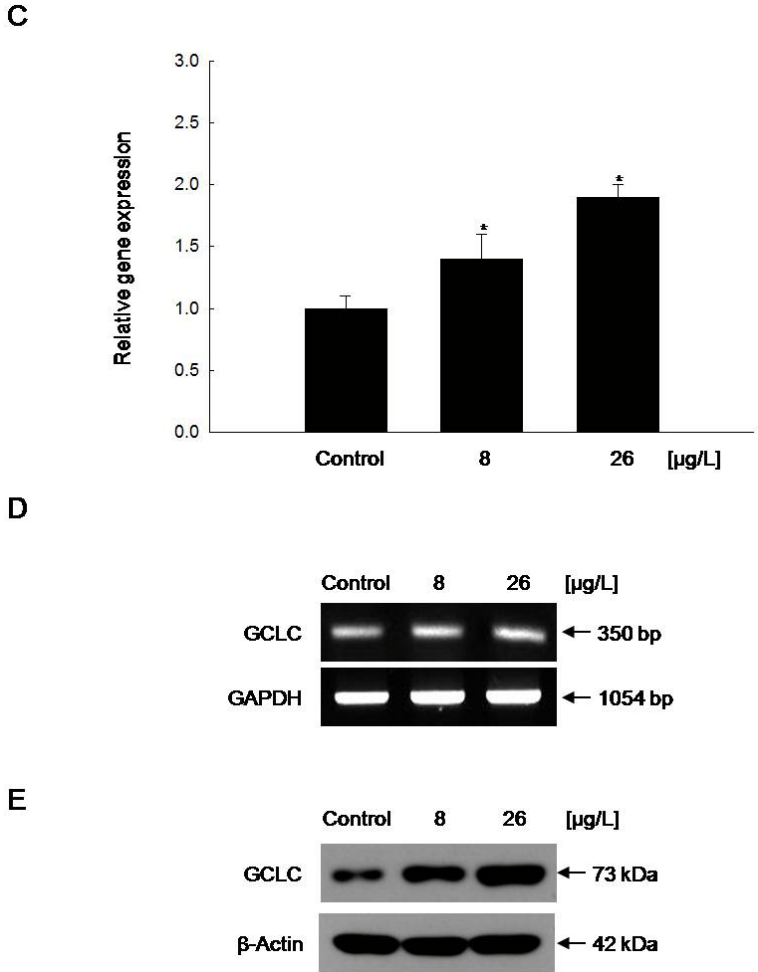
Vanadyl sulfate increases cellular GSH level and induces catalytic subunit of glutamate cysteine ligase (GCLC) expression. Cellular GSH levels were detected using (**A**) a colorimetric assay kit and (**B**) confocal microscopy after CMAC staining. GCLC mRNA gene expression was measured by (**C**) real-time PCR and (**D**) RT-PCR; (**E**) Cell lysates were electrophoresed, and GCLC protein was subsequently detected using a specific antibody. * Significantly different from the control group (*P* < 0.05).

Vanadyl sulfate treatment at 8 and 26 μg/L effectively increased cellular GSH level to 126 and 142 μM respectively compared to 110 μM in DDW treatment (control) (Figure 3A). Confocal microscopy showed increases in the blue fluorescence intensity of cellular GSH in VOSO_4_-treated cells compared to DDW-treated cells (control) (Figure 3B). We also studied GCLC mRNA and protein expression in response to vanadyl sulfate, using real time-PCR and RT-PCR as well as Western blot analysis, respectively. Compared to DDW group (control), vanadyl sulfate treatment at 8 and 26 μg/L induced GCLC mRNA and protein expression (Figure 3C–E).

### 2.4. Increased GCLC Gene Transcription Following the Induction of Nuclear Translocation of Nrf2 by Vanadyl Sulfate

**Figure 4:**
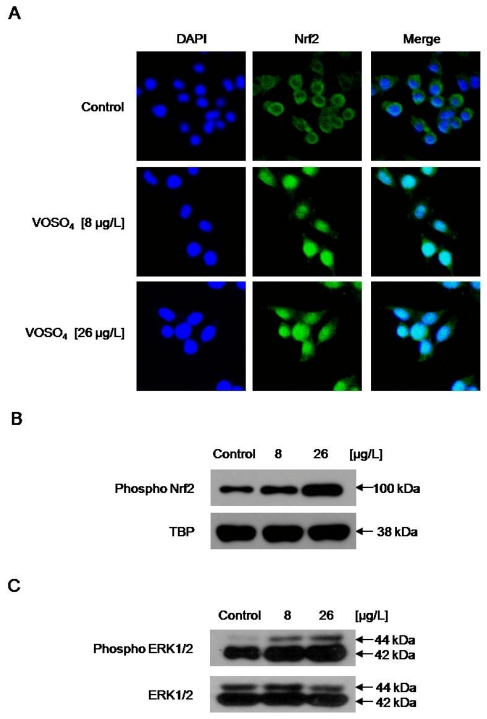
Vanadyl sulfate induces the activation of erythroid transcription factor NF-E2 (Nrf2) and extracellular regulated kinase (ERK). (**A**) Confocal images of cells stained with fluorescein isothiocyanate-conjugated secondary antibody after incubation of the cells with Nrf2 primary antibody show the location of Nrf2 (green). 4′,6-diamidino-2-phenylindole (DAPI) staining indicates the location of the nucleus (blue). The merged image in VOSO_4_-treated cells shows the nuclear location of Nrf2 protein; (**B**) Nuclear extracts were prepared and subjected to Western blotting using a phospho Nrf2-specific antibody; (**C**) Cell lysates were electrophoresed and subjected to Western blotting to detect phospho ERK1/2 and ERK1/2 using their respective specific antibodies.

Since the transcription factor Nrf2 regulates ARE-driven GCLC gene expression [5,14], we examined whether vanadyl sulfate is able to activate Nrf2 in association with the up-regulation of GCLC. VOSO_4_ treatment increased both the nuclear accumulation of Nrf2 protein from the cytosol (Figure 4A) and the level of the active form of protein (phospho Nrf2) (Figure 4B). To further elucidate the upstream signaling pathway involved in VOSO_4_-mediated Nrf2 activation, we examined the activation of ERK, a major signaling enzyme involved in the nuclear translocation of activated Nrf2 [15]. Vanadyl sulfate-mediated ERK activation was assessed on Western blot using phospho-specific antibody against ERK. As shown in Figure 4C, VOSO_4_ treatment caused an increase in ERK phosphorylation.

## 3. Experimental Section

### 3.1. Reagents

Jeju ground water containing the vanadium components; S1 (vanadium: 8.0 ± 0.9 μg/L) and S3 (vanadium: 26.0 ± 2.0 μg/L, Na^+^: 5.1 ± 1.0 mg/L, Ca^2+^: 3.4 ± 0.5 mg/L, Mg^2+^: 3.0 ± 1.0 mg/L, K^+^: 3.0 ± 0.5 mg/L) was provided by the Jeju special self-governing province development corporation (Jeju, Korea). Vanadyl sulfate (VOSO_4_), 5,5-dimethyl-1-pyrroline-*N*-oxide (DMPO) and 2′,7′-dichlorodihydrofluorescein diacetate (DCF-DA) were purchased from Sigma Chemical Company (St. Louis, MO, USA). The catalytically active subunit of glutamate-cysteine ligase (GCLC) antibody was purchased from Thermo Scientific (Fremont, CA, USA). The phospho ERK1/2 and ERK1/2 antibodies were purchased from Santa Cruz Biotechnology (Santa Cruz, CA, USA), phospho Nrf2 and Nrf2 antibodies from Epitomics Inc. (Burlingame, CA, USA). Tert-butoxycarbonyl-Leu-Met-7-amino-4-chloromethylcoumarine (CMAC) was purchased from Molecular Probes (Eugene, OR, USA). All other chemicals and reagents were of analytical grade.

### 3.2. Cell Culture

Human Chang liver cells were obtained from the American Type Culture Collection (Rockville, MD, USA) and maintained at 37 °C in an incubator with a humidified atmosphere of 5% CO_2_ in air. Cells were cultured with RPMI 1640 containing distilled deionized water (DDW), Jeju ground water (S1 and S3), or vanadyl sulfate supplemented with 0.1 mM non-essential amino acids, 10% heat-inactivated fetal calf serum, streptomycin (100 μg/mL), and penicillin (100 units/mL).

### 3.3. Intracellular GSH Measurement

Intracellular GSH content was measured using a commercial colorimetric assay kit, GSH-400, from OXIS International (Portland, OR, USA). Cells were harvested and then homogenized in a metaphosphoric working solution and then centrifuged. Fifty microliters of R1 solution containing a chromogenic reagent in HCl were added to 900 μL of supernatant, followed by gentle vortex mixing. Fifty microliters of R2 solution (30% NaOH) were then added and the mixtures were incubated at 25 ± 3 °C for 10 min. After centrifugation, the absorbance of the clear supernatant was measured at 400 nm. In addition, image analysis of the intracellular GSH level was assessed by incubating the cells with 5 μM C MAC, a GSH-sensitive fluorescent dye, for 30 min in the dark. CMAC fluorescence images were analyzed using a Zeiss Axiovert 200 inverted microscope at an excitation wavelength of 351 nm and an emission wavelength of 380 nm [16].

### 3.4. Detection of Superoxide Anion

Xanthine/xanthine oxidase was used to generate superoxide anion, which was then reacted with a nitrone spin trap DMPO. The DMPO/·OOH adducts were detected using an electron spin resonance (ESR) spectrometer (JEOL, Tokyo, Japan) [17,18]. ESR signaling was recorded 5 min after the addition of 20 μL each of xanthine oxidase (0.25 U/mL), xanthine (5 mM), and DMPO (1.5 M), and either DDW or VOSO_4_. The parameters of the ESR spectrometer were as follows: magnetic field, 336 mT; power, 5.00 mW; frequency, 9.4380 GHz; modulation amplitude, 0.2 mT; gain, 500; scan time, 0.5 min; scan width, 10 mT; time constant, 0.03 s; and temperature, 25 °C.

### 3.5. Detection of Hydroxyl Radical

Hydroxyl radical was generated by the Fenton reaction (H_2_O_2_ + FeSO_4_) and then reacted with DMPO. The resultant DMPO/·OH adducts were detected using an ESR spectrometer [19,20]. ESR signaling was recorded 2.5 min after the addition of 20 μL each of 0.3 M DMPO, 10 mM FeSO_4_, 10 mM H_2_O_2_, and either DDW or VOSO_4_. The parameters of the ESR spectrometer were as follows: magnetic field, 336 mT; power, 1.00 mW; frequency, 9.4380 GHz; modulation amplitude, 0.2 mT; gain, 200; scan time, 0.5 min; scan width, 10 mT; time constant, 0.03 s; and temperature, 25 °C.

### 3.6. Measurement of Intracellular Reactive Oxygen Species (ROS)

Cells were treated with 25 μM DCF-DA and the fluorescence of 2′,7′-dichlorofluorescein was detected using a Perkin Elmer LS-5B spectrofluorometer and a flow cytometer (Becton Dickinson, Mountain View, CA, USA) [21]. The image analysis for the generation of intracellular ROS was analyzed by seeding cells on a cover-slip-loaded six-well plate at 2 × 10^5^ cells/well. DCF-DA (100 μM) was added to each well followed by incubation for an additional 30 min at 37 °C. The stained cells were washed with phosphate buffered-saline (PBS) and then mounted onto microscope slide in mounting medium (DAKO, Carpinteria, CA, USA). Microscopic images were collected using the laser scanning microscope 5 PASCAL program (Carl Zeiss, Jena, Germany) of a confocal microscope.

### 3.7. Real Time-Polymerase Chain Reaction (PCR)

Total RNA was isolated from cells using Trizol (GibcoBRL, Grand Island, NY, USA). Quantitative real time-PCR was performed in 96-well optical plates with an iQ^TM5^ multicolor real time-PCR detection system (Bio-Rad, Hercules, CA, USA). The primer pairs (Bionics, Seoul, South Korea) were as follows (forward and reverse, respectively): GCLC, 5′-AGTTCAATACAGTTGAGG-3′ and 5′-TACTGATCCTATAGTTAT-3′; and GAPDH, 5′-AAGGTCGGAGTCAACGGATTT-3′; and 5′-GCAGTGAGGGTCTCTCTCCT-3′. The PCR mixture contained 10 μL of 2× SYBR qPCR SuperMix Universal kit (Invitrogen, CA, USA), 10 μM each of the forward and reverse primers, and 1 μL of diluted template cDNA (10 ng). Fluorescence was measured at the end of each cycle to determine the amount of the PCR products. The point at which the SYBR fluorescent signal reached statistical significance above background was defined as the cycle threshold (CT), the optimal value of which was chosen automatically. Transcript quantities represented the expression levels of target genes and were determined relative to those of reference genes using the following equation, based on the gene expression CT difference method [22]: $\text{relative expression level}=\frac{{{\text{E}}_{\text{HKG}}}^{\text{CTHKG}}}{{{\text{E}}_{\text{GOI}}}^{\text{CTGOI}}}$, where E_HKG_ and E_GOI_ are the PCR efficiencies, and CT_HKG_ and CT_GOI_ are the threshold cycles for the reference housekeeping gene (GAPDH) and the gene of interest (GCLC), respectively.

### 3.8. Reverse Transcription Polymerase Chain Reaction (RT-PCR)

Total RNA was isolated from cells using Trizol (GibcoBRL). RT-PCR for GCLC was carried out with the same GCLC primer pairs (Bionics) used for real time-PCR under the following conditions: 40 cycles at 94 °C for 15 s, 53 °C for 30 s, and at 72 °C for 30 s, with a final extension for 5 min at 72 °C. The RT-PCR conditions for housekeeping gene GAPDH were: 30 cycles at 94 °C for 15 s, 60 °C for 30 s, and at 72 °C for 30 s, with a final extension for 5 min at 72 °C. Amplified products were resolved on 1% agarose gels, stained with ethidium bromide, and photographed under UV light.

### 3.9. Western Blotting Analysis

Cells were harvested, washed twice with PBS, lysed on ice for 30 min in 100 μL of lysis buffer [120 mM NaCl, 40 mM Tris (pH 8), 0.1% NP 40], and then centrifuged at 13,000 × g for 15 min. The supernatants were collected from the lysates and the protein concentrations were determined. Aliquots of the lysates (40 μg of protein) were boiled for 5 min and electrophoresed in a 10% SDS-polyacrylamide gel. The proteins in the gels were transferred onto nitrocellulose membranes, which were then incubated with the primary antibody and then with the secondary antibody conjugated to horseradish peroxidase (Pierce, Rockford, IL, USA). Protein bands were detected using an enhanced chemiluminescence Western blotting detection kit (Amersham, Little Chalfont, Buckinghamshire, UK) and then exposed to X-ray film.

### 3.10. Immunocytochemistry

Cells plated on coverslips were fixed with 4% paraformaldehyde for 30 min, permeabilized with 0.1% Triton X-100 in PBS for 2.5 min, treated with blocking medium (3% bovine serum albumin in PBS) for 1 h, and then incubated with Nrf2 antibody diluted in blocking medium for 2 h. Immune-reacted primary Nrf2 antibody was detected following for 1 h incubation with a 1:500 dilution of FITC-conjugated secondary antibody (Jackson ImmunoResearch Laboratories, West Grove, PA, USA). The stained cells were washed and then mounted onto microscope slides in mounting medium with DAPI (Vector, Burlingame, CA, USA). Images were collected using the LSM 510 program on a Zeiss confocal microscope.

### 3.11. Statistical Analysis

All measurements were made in triplicate (*n* = 3), and all values are the means ± standard error. The data were subjected to analysis of variance (ANOVA) using the Tukey test.

## 4. Conclusions

Electrolytes and the balance of minerals such as vanadium, magnesium, chromium, and zinc are essential for the body’s ability to maintain homeostasis and thus for its overall health. Vanadium compounds reportedly exert antioxidant effects [10,11] and, by down-regulating inducible nitric oxide synthase, as shown in rat colon, confer protection against genotoxicity and carcinogenesis [23]. In another study, vanadate treatment restored the decreased activity of antioxidant enzymes and the altered level of plasma lipid peroxide in diabetic rats [24]. Our results suggest that vanadyl sulfate exerts scavenging effects on superoxide anion, hydroxyl radical and intracellular ROS. However, vanadium is also a catalyst that can induce ROS generation *in vitro* and lipid peroxidation and oxidative damage in experimental models [25]. These contradictory effects of vanadium compounds, as antioxidants and pro-oxidants, have been ascribed to differences in the dose and the experimental conditions [26].

Glutathione is required for the maintenance of cell integrity, based on its reducing properties and its participation in cell metabolism [1]. The administration of vanadyl sulfate to diabetic rat was shown to increase brain GSH content, thus protecting cellular proteins against oxidation through the glutathione redox cycle [27]. Our results indicate that vanadyl sulfate stimulates GSH synthesis through the up-regulation of GCLC expression. The maintenance of cellular GSH level involves *de novo* and salvage pathways. Ha *et al.* reported that zinc increases the GSH level through *de novo* synthesis [28], but it is still not clear which pathway is activated by vanadyl sulfate. The redox-sensitive transcription factor Nrf2 is important in the transcriptional regulation of GCL, and thus in regulating GSH level [29]. GCL activity is associated with the nuclear localization of Nrf2. Oxidative stress or electrophilic compounds have been shown to modify the thiol groups of Kelch-like ECH-associated protein 1 (Keap 1) or Nrf2 [30], and/or activate upstream signaling kinase [15,31,32], resulting in the dissociation of Nrf2 from Keap1. Dissociation allows Nrf2 to translocate to the nucleus, where its accumulation leads to an increase in GCL transcription. In our system, treatment of the cells with vanadyl sulfate induced the nuclear translocation and accumulation of Nrf2. Leong *et al.* demonstrated that schisandrin B activates ERK, which is followed by an enhancement in Nrf2 nuclear translocation and the glutathione antioxidant response [15,33]. In our system, vanadyl sulfate-treated cells expressed higher level of phosphorylated nuclear Nrf2, which was presumably mediated by the phosphorylation activity of activated ERK.

In conclusion, our study suggests that the vanadium compounds contained in Jeju ground water increased GSH levels through a mechanism in which vanadyl sulfate triggers redox-sensitive ERK/Nrf2 signaling and, ultimately, GCLC expression.
